# Supplementary material for: Testing for a facultative locomotor mode in the acquisition of archosaur bipedality
Source: R Soc Open Sci. 2019 Jul 17;6(7):190569. doi: 10.1098/rsos.190569 (PMC6689609; doi:10.1098/rsos.190569)
Supplement: Table of locomotor diagnoses [file rsos190569supp1.docx]

| Taxon | Source (Description where applicable;  Diagnostic paper if different to original) | Locomotor  mode | Text or diagram based diagnosis and supporting statement |
| --- | --- | --- | --- |
| Acerosodontosaurus_piveteaui | (Currie, 1980); (Bickelmann, Mü and Reisz, 2009) | OQ | Text – “aquatic” therefore quadruped based on assumptions in the manuscript. |
| Aenigmastropheus_parringtoni | (Ezcurra, Scheyer and Butler, 2014) | OQ | Diagram – text states “k value … higher than those observed in aquatic and semi-aquatic animals, supporting a terrestrial mode of life”. |
| Aetosauroides_scagliai | (Casamiquela, 1960); (Desojo *et al.*, 2013) | OQ | Text – “a clade of obligately quadrupedal, heavily armoured pseudosuchians”. |
| Aetosaurus_ferratus | (Schoch, 2007); (Desojo *et al.*, 2013) | OQ | Text – as above. |
| Alligator_mississippiensis | (Daudin 1801); (Allen *et al.*, 2010) | OQ | Text – “﻿Extant crocodylians are the only known living tetrapods to use nearly the full range of recognized quadrupedal terrestrial locomotion patterns”. |
| Allosaurus_fragilis | (Marsh, 1877); (Farlow *et al.*, 2000) | OB | Text – “Theropods were erect, digitigrade, striding bipeds”. |
| Amotosaurus_rotfeldensis | (Fraser and Rieppel, 2006) | OQ | Text – semi-aquatic “juveniles of *T. antiquus* were at least partly terrestrial. By contrast, they became fully marine when adult”. Juvenile *T. antiquus* specimens were reassigned to *Amotosaurus rotfeldensis*. Also, “clearly a tanystropheid”, a group which exhibits an unusual, long-necked morphology. |
| Archeopelta_arborensis | (Desojo, Ezcurra and Schultz, 2011) | OQ | Diagram – within the text, a possible aquatic lifestyle is alluded to, or a terrestrial foraging lifestyle. |
| Arizonasaurus_babbitti | (Welles, 1947); (Nesbitt, 2005) | OQ | Diagram – no caudal vertebrae or limb bones. Diagnosed based on a reconstruction. |
| Asilisaurus_kongwe_combined | (Nesbitt *et al.*, 2010) | OQ | Text – “a member of the Silesauridae”, “Silesaurids were diverse … with … a quadrupedal stance”. |
| Azendohsaurus_madagaskarensis | (Flynn *et al.*, 2010); (Nesbitt *et al.*, 2015) | OQ | Diagram – the text contains very thorough descriptions of many specimens that suggest quadrupedality without explicitly stating it. “an elongated neck, a short, stocky tail, robust limbs”. |
| Batrachotomus_kupferzellensis | (Gower, 1999); (Gower and Schoch, 2009) | OQ | Diagram – largely complete post-crania with a complete skull. Diagram is in a quadrupedal pose. |
| Bentonyx_sidensis | (Langer *et al.*, 2010); (Ezcurra, Montefeltro and Butler, 2016) | OQ | Text – “rhynchosaurs were bulky, herbivorous and quadrupedal animals” |
| Chanaresuchus_bonapartei | (Romer, 1971b); (Romer, 1972a) | OQ | Text – “I have restored the animal as a quadruped”. However, Romer tentatively debates facultative bipedality as a consequence of an amphibious lifestyle. As this animal is a terminal taxa deep in a OB section of the tree, a character state change here would not affect the rest of the analysis. |
| Chasmatosaurus_yuani | (Young, 1936); (Charig and Reig, 1970) | OQ | Text – “they were undoubtedly quadrupedal, despite the typical archosaurian limb disparity”. |
| CM_73372 | (Weinbaum, 2013) | OB | Text - ﻿CM-73372 is used for informing the anatomy of *Postosuchus*, deemed “an obligate biped” based on “limb proportions … the size of the manus … and the highly reduced nature of the digits and vertebral measurements”, thus is considered a biped here, though this is contentious. Removed in sensitivity analysis. |
| Coelophysis_bauri | (Cope, 1887) | OB | Text – also in many studies since. Unequivocal derived theropod, thus bipedal. |
| Cteniogenys_spp | (Gilmore, 1928); (Evans, 1990) | OQ | Text – many aquatic features, thus diagnosed as quadrupedal. |
| Dibothrosuchus_elaphros | (Simmons, 1965); (Wu, 1986; Wu and Chatterjee, 1993) | OQ | Text – “The limbs are long, slender and adapted for quadrupedal terrestrial gait”. |
| Dilophosaurus_wetherelli | (Welles, 1954) | OB | Text – theropod dinosaur, bipedal. |
| Dimorphodon_macronyx | (Buckland, 1829); (Frigot, 2018) | OQ | Text – “Dimorphodon was an obligate quadruped.” However, it should be noted that pterosaur posture and gait is contentious, with support for both bipedal and quadrupedal stances prevalent in the literature (Padian, 2008; Witton, 2015) |
| Dongusuchus_efremovi | (Sennikov, 1988); (Nesbitt *et al.*, 2017) | OQ | Diagram – however, the text states that Aphanosaurs were “long-necked, non-cursorial” and “so more like stem-archosaurs than later avemetatarsalians”, which all suggest obligate quadrupedality. |
| Doswellia_kaltenbachi | (Weems, 1980), (Dilkes and Sues, 2009); (Sues, Desojo and Ezcurra, 2013) | OQ | Text - in Weems (1980): “front limbs almost certainly functioned as strong walking structures”, facultative bipedality is mentioned and dismissed as a possibility based on pelvic osteology. Sues et al (2013) suggests semi-aquatic lifestyle, hence OQ. |
| Dromomeron_gregorii | (Sterling J. Nesbitt, Irmis, *et al.*, 2009); (Fechner, 2009) | FB | Text – anatomical analysis, identifies *Dromomeron* as facultatively bipedal |
| Dromomeron_romeri | (Irmis *et al.*, 2007); (Fechner, 2009) | FB | Text – anatomical analysis, identifies *Dromomeron* as facultatively bipedal |
| Effigia_okeeffeae | (Nesbitt and Norell, 2006); (Nesbitt, 2007) | OB | Text – “﻿The convergences suggest that a ‘theropod dinosaur body plan’ developed in a group of crocodile-line archosaurs before it appeared in theropod dinosaurs.”. This indicates bipedality, supported by a diagram in a bipedal pose. |
| Efraasia_minor | (Huene, 1908); (Galton, 1973; Kubo and Kubo, 2012) | FB | Text – anatomical analysis indicated *Efraasia* exists in a facultatively bipdal region of Kubo and Kubo’s Quadrupedality Index |
| Eoraptor_lunensis | (Sereno *et al.*, 1993); (Sereno, Mar Inez and Alcober, 2013) | OB | Text – basal sauropodomorph, limb proportions “consistent with bipedal posture at speed”, but does not mention quadrupedality, therefore classified bipedal. Additionally, represented as a biped in the skeletal reconstruction. |
| Erythrosuchus_africanus | (Broom, 1905); (Ezcurra, Butler and Gower, 2013) | OQ | Diagram – text states that no detailed locomotor studies have been performed, previous studies have alluded to a possible semi-aquatic lifestyle. |
| Eudimorphodon_ranzii | (Zambelli, 1973); (Witton, 2015) | OQ | Text – See Dimorphodon for discussion regarding pterosaur posture |
| Euparkeria_capensis | (Broom, 1913); (Ewer, 1965) | FB | Text – various morphological analyses in the same vein as “this contrast between fore and hind limbs is strongly suggestive of facultative bipedalism”. |
| Garjainia_madiba_combined | (Gower *et al.*, 2014) | OQ | Diagram – life reconstruction by paleontologist and paleoartist Mark Witton. Garjainia prima is diagnosed by text in (Ezcurra *et al.*, 2018), which is a close relative. |
| Garjainia_prima | (Ochev, 1958); (Ezcurra *et al.*, 2018) | OQ | Text – “﻿The erythrosuchids were quadrupedal, probably sprawling and bulky, predatory archosauromorphs”. |
| Gephyrosaurus_bridensis | (Evans, 1980); (Evans, 1981) | OQ | Text – ﻿“Gephyrosaurus shows no structural changes in this direction and it was probably a swift quadruped” . |
| Gracilisuchus_stipanicicorum | (Romer 1972) | FB | Text – “It is possible that the normal pose of *Gracilisuchus* was a quadrupedal one; however I feel sure that, if pressed, this animal was able to run in the bipedal manner in which I have restored it.”. This has been reiterated in works completed since, e.g. (Kubo and Kubo, 2012). |
| Gualosuchus_reigi | (Romer, 1971b) | OQ | Text – Semi-aquatic: “The general structure of these four genera is strongly suggestive of amphibious habits”. |
| Herrerasaurus_ischigualastensis | (Reig, 1963); (Sereno and Novas, 1992) | OB | Text – “*Herrerasaurus*, a primitive theropod, was an agile, bipedal predator”. |
| Hesperosuchus_agilis | (Colbert, 1952) | FB | Text – “It is probable that *Hesperosuchus* … also utilised both the quadrupedal and bipedal types of locomotion.” |
| Heterodontosaurus_tucki | (Crompton and Charig, 1962); (Pontzer, Allen and Hutchinson, 2009) | OB | Text – “… in the small, presumably active bipeds, *Hesperosuchus*, *Compsognathus* and *Velociraptor*”. |
| Jaxtasuchus_salomoni | (Schoch and Sues, 2014) | OQ | Text – semi-aquatic: “*Jaxtasuchus* has an elongate body form  with a long tail and proportionately small limbs, which, together with its extensive dorsal armour, lent it a rather crocodile-like appearance that, in turn, may reflect an at least semi-aquatic mode of life”. |
| Jesairosaurus_lehmani | (Jalil, 1997) | FB | Text – “The elongation of the hindlimb associated with the shortening of the neck , in comparison with other prolacertiforms, might be correlated with facultative bipedalism in Jesairosaurus.”. |
| Lagerpeton_chanarensis | (Romer, 1971a); (Fechner, 2009) | FB | Text – anatomical analysis indicates facultative bipedalism, “The transversely broad pelvis, the adductor-controlled postural support and especially the intermediate hindlimb posture clearly indicate that obligate bipedal locomotion has to be ruled out for Lagerpeton.” |
| Lesothosaurus_diagnosticus | (Galton, 1978); (Bates *et al.*, 2012, 2015; Baron, Norman and Barrett, 2016) | OB | Text – anatomical, 3D computational modelling. Correction paper (2015) confirms that major findings from original study (2012) are not changed. |
| Litargosuchus_leptorhynchus | (Clark and Sues, 2002); (Gow and Kitching, 1988) | FB | Text – previous analysis when the specimen was assigned to *Pediticosaurus* state that “*Pediticosaurus* was unquestionably bipedal at speed”. Though the specimen has been reassigned, the available material has not changed, and locomotor mode has not been reassessed. |
| Longosuchus_meadei | (Sawin, 1947); (Desojo *et al.*, 2013) | OQ | Text – “All aetosaurs were obligate quadrupeds”. *Longosuchus* is alternatively named *Typothorax.* |
| Lotosaurus_adentus | (Zhang, 1975); (Hagen *et al.*, 2018) | OQ | Text – assortment of anatomical features “indicative of quadrupedality”. |
| Machaeroprosopus_pristinus | (Mehl, 1928); (Stocker and Butler, 2013) | OQ | Text – “They were quadrupedal” in reference to phytosaurs generally, this animal is included in their list of phytosaurs. |
| Macrocnemus_bassanii | (Nopcsa, 1930); (Rieppel, 1989) | FB | Text – “A functional analysis of the appendicular skeleton suggests predominantly terrestrial habits, perhaps even facultative bipedalism during rapid locomotion”. |
| Marasuchus_lilloensis | (Romer 1972b); (Sereno and Arcucci, 1994) | OB | Text – “﻿Two small bipedal archosaurs in the Middle Triassic  Los Chafiares fauna, Lagerpeton chanarensis and Marasuchus lilloensis, provide the best available evidence of ornithodirans that predate the Late Triassic America radiation of dinosaur”. Based on anatomical analysis and reconstruction of a near complete specimen. |
| Mesosuchus_browni | (Watson, 1912b); (Dilkes, 1998) | OQ | Diagram – skeletal reconstruction in quadrupedal stance based on a number of different specimens |
| Nicrosaurus_kapffi | (Meyer, 1860); (Kimmig, 2013; Stocker and Butler, 2013) | OQ | Text – As for all phytosaurs, “They were quadrupedal” and *Nicrosaurus* is included in the list of phytosaurs in the Stocker and Butler (2013) analysis |
| Noteosuchus_colletti | (Watson, 1912a); (Ezcurra, 2016; Ezcurra, Montefeltro and Butler, 2016) | OQ | Text – “rhynchosaurs were bulky, herbivorous and quadrupedal animals”. |
| Nundasuchus_songeaensis | (Nesbitt *et al.*, 2014) | OQ | Diagram – relatively complete hindlimb, partial forelimb, partial postcrania. |
| Ornithosuchus_longidens | (Huxley, 1877); (von Baczko and Ezcurra, 2013; von Baczko and Desojo, 2016) | OQ | Text – “*Ornithosuchus* might have been facultative bipedal animals, acquiring an upright posture during fast gaits.” was a previous interpretation, but this has been more recently overwritten by “Ornithosuchids are terrestrial quadrupedal carnivorous archosaurs”. There is scope for considering *Ornithosuchus* a facultative biped. |
| Pamelaria_dolichotrachela | (Sen, 2003) | OQ | Text – “The reconstructed skeleton … suggests that it was a quadrupedal walker.”. |
| Parasuchus_hislopi | (Lydekker, 1885); (Chatterjee, 2001; Stocker and Butler, 2013) | OQ | Text – As for all phytosaurs, “They were quadrupedal” and *Parasuchus* is included in the list of phytosaurs in the Stocker and Butler (2013) analysis |
| Petrolacosaurus_kansensis | (Lane, 1945); (Peabody, 1952) | OQ | Text – “Petrolacosaurus was terrestrial, fully quadrupedal in gait, perhaps arboreal, and that it was capable of rapid locomotion.”. |
| Planocephalosaurus_robinsonae | (Fraser, 1982); (Fraser and Walkden, 1984) | OQ | Text – “The small limb disparity in quadrupedal eosuchians, such as *Planocephalosaurus*” |
| Plateosaurus_engelhardti | (Meyer, 1837); (Mallison, 2010) | OB | Text – “Here, I present evidence, derived from a detailed mounting of a 3D digital skeleton and a computer-aided engineering assessment of a digital 3D model of the living animal, that *Plateosaurus* was indeed an obligate biped.”. |
| Polonosuchus_silesiacus | (Sulej, 2005) | OQ | Text – “﻿The new species was  probably similar to *P. kirkpatricki* in general view. *P. kirkpatricki* was a medium-sized (3 m long) heavily built rauisuchid, a large- skulled and short-necked, non-cursorial quadruped”. |
| Poposaurus_gracilis_holotype | (Mehl, 1915); (Gauthier *et al.*, 2011; Schachner, Manning and Dodson, 2011) | OB | Text – “﻿This animal represents the most complete poposauroid skeleton known to date, and one of the most complete bipedal basal archosaurs yet discovered”. |
| Poposaurus_gracilis_yale | (Mehl, 1915); (Gauthier *et al.*, 2011; Schachner, Manning and Dodson, 2011) | OB | Text - “﻿This animal represents the most complete poposauroid skeleton known to date, and one of the most complete bipedal basal archosaurs yet discovered” |
| Postosuchus_alisonae | (Peyer *et al.*, 2008); (Weinbaum, 2013) | OB | Text – “evidence … suggests that *Postosuchus* may have been habitually bipedal”, “Analysis of the postcranial skeleton of *Postosuchus* suggests it may have been an obligate biped” based on anatomical features. The analysis in this study is based on mulitiple *Postosuchus* specimens, so we apply it to both *P. alisonae* and *P. kirkpatricki* here. |
| Postosuchus_kirkpatricki | (Chatterjee, 1985); (Weinbaum, 2013) | OB | Text – As above. |
| Prestosuchus_chiniquensis | (Huene, 1942); (Liparini and Schultz, 2013) | FB | Text – “*Prestosuchus chiniquensis* may be a quadruped, facultative biped, with semi-erect to parasagittal limb postures.”. |
| Prolacerta_broomi | (Parrington, 1935); (Gow, 1975) | FB | Text – “﻿Prolacerta was clearly a bipedal runner with a large tail to counter- balance the weight of the body.”. MS makes it clear that this is a running adaptation throughout and is reconstructed diagrammatically as a quadruped, so facultative bipedality is used. |
| Proterochampsa_barrionuevoi | (Reig, 1959); (Trotteyn, Arcucci and Raugust, 2013) | OQ | Text – semi-aquatic, stated in the Paleobiology section of this work. Also reconstructed diagrammatically as a quadruped. We use this analysis for both species of Proterochampsa. |
| Proterochampsa_nodosa | (Barberena, 1982); (Trotteyn, Arcucci and Raugust, 2013) | OQ | Text – As above. |
| Proterosuchus | (Broom, 1903) | OQ | Text – Semi-aquatic: “Proterosuchids are diapsids that probably had a predominantly sprawling gait”, “mostly aquatic, predacious reptiles living in ponds, lakes and rivers, using swimming as their main form of locomotion”, “similar lifestyle to that of extant crocodiles”. |
| Proterosuchus_alexanderi | (Hoffman, 1965); (Ezcurra, Butler and Gower, 2013) | OQ | Text – As above |
| Proterosuchus_fergusi | (Broom, 1903); (Ezcurra, Butler and Gower, 2013) | OQ | Text – As above |
| Proterosuchus_goweri | (Ezcurra and Butler, 2015); (Ezcurra, Butler and Gower, 2013) | OQ | Text – As above |
| Protorosaurus_speneri | (Meyer, 1832); (Gottman-Quesada and Sander, 2009) | OQ | Text – “*Protosaurus* is a quadrupedal reptile.” |
| Protosuchus_richardsoni | (Brown, 1933); (Colbert, Mook and Brown, 1951) | FB | Text – “Protosuchus probably had a varied mode of locomotion, partially bipedal and partially quadrupedal.”. |
| Pseudochampsa_ischigualastensis | (Trotteyn, Arcucci and Raugust, 2013); (Trotteyn and Ezcurra, 2014) | OQ | “Proterochampsids are crocodile-like, probably semi-aquatic, quadrupedal archosauriforms”. |
| Qianosuchus_mixtus | (Li *et al.*, 2006) | OQ | Text – semi-aquatic: ﻿“*Q. mixtus* is unique among Triassic archosaurians in having a mosaic of specializations for both aquatic and terrestrial ways of life.”. |
| Rauisuchus_triradentes | (Huene, 1942); (Nesbitt *et al.*, 2013; Lautenschlager and Rauhut, 2015) | OQ | Text – “Mid- to large-sized quadrupedal predators” in reference to Rauisuchidae, which *Rauisuchus* is member of. Also a quadrupedal diagram in Lautenschlager & Rahut (2015). |
| Rhynchosaurus_articeps | (Owen, 1842); (Benton, 1990) | OQ | Diagram – however, with a lot of text describing the posture as “semi-erect hindlimb” and “partly sprawling forelimb” in a “‘fast walking’ pose” |
| Riojasuchus_tenuisceps | (Bonaparte, 1969); (von Baczko and Desojo, 2016) | OQ | Text – “Ornithosuchids are terrestrial quadrupedal carnivorous archosaurs”. Previous arguments applied to *Ornithosuchus* could apply here. A detailed biomechanical analysis of this largely complete specimen would be insightful. |
| Sacisaurus_agudoensis | (Ferigolo and Langer, 2007; Langer and Ferigolo, 2013) | OB | Diagram – However, this animal was first described as an early ornithischian rather than a non-dinosaurian dinosauromorph, so this classification is contentious. Given how this animal’s bipedality is an end result in a complete transition OQ > FB > OB, the lack of clarity surrounding this animal is paramount to examining the transition in detail. |
| Saturnalia_tupiniquim | (Langer *et al.*, 1999); (Bronzati *et al.*, 2017) | FB | Text – “﻿*Saturnalia* *tupiniquim* is a facultative biped”. |
| Saurosuchus_galilei | (Reig, 1959); (Nesbitt *et al.*, 2013) | OQ | Text – “Mid- to large-sized quadrupedal predators” in reference to Rauisuchidae, which *Saurosuchus* is member of. |
| Scutellosaurus_lawleri | (Colbert, 1981); (Breeden, 2016) | OB | Text – “﻿A small (~1 m) bipedal thyreophoran ornithischian dinosaur” |
| Shansisuchus_shansisuchus | (Young, 1964); (Ezcurra *et al.*, 2018) | OQ | Text – Erythrosuchid, “The erythrosuchids were quadrupedal, probably sprawling and bulky, predatory archosauromorphs”. |
| Shuvosaurus_inexpectatus | (Chatterjee, 1993); (Nesbitt, Irmis and Parker, 2007) | OB | Text – “﻿The convergences suggest that a ‘theropod dinosaur body plan’ developed in a group of crocodile-line archosaurs before it appeared in theropod dinosaurs.”. Indicative of a bipedal locomotor mode. |
| Silesaurus_opolensis | (Dzik, 2003); (Piechowski and Dzik, 2010) | FB | Text – “﻿This suggests a greater load on the pelvic girdle and the ability of Silesaurus to run bipedally on occasion”. |
| Sillosuchus_longicervix | (Alcober and Parrish, 1997); (Parker and Nesbitt, 2013) | OB | Text – “We therefore hypothesize that *S. longicervix* was also bipedal”. |
| Simoedosaurus_lemoinei | (Gervais, 1877); (Matsumoto and Evans, 2010) | OQ | Text – aquatic therefore OQ, mentioned repeatedly throughout this paper. |
| Smilosuchus_gregorii | (Camp, 1930); (Stocker and Butler, 2013) | OQ | Text – “They were quadrupedal” in reference to phytosaurs generally, this animal is included in their list of phytosaurs.”. |
| Sphenosuchus_acutus | (Haughton, 1915); (Walker, 1990) | OQ | Text – “There is little doubt that S*phenosuchus* was quadrupedal.”. |
| Spondylosoma_absconditum | (Huene, 1942); (Nesbitt *et al.*, 2017) | OQ | Diagram – in a diagram with other Aphanosaurs. |
| Stagonolepis_robertsoni | (Agassiz, 1844); (Desojo *et al.*, 2013) | OQ | Text – “a clade of obligately quadrupedal, heavily armoured pseudosuchians”. |
| Staurikosaurus_pricei | (Colbert, 1970) | OB | Text – “﻿A bipedal saurischian of small size, with strong hind limbs  and small forelimbs.”. |
| Tanystropheus_longobardicus | (Bassani, 1886); (Renesto, 2005) | OQ | Text – semi-aquatic or shoreline taxa with an extreme neck morphology. Plenty of discussion about counterbalancing the body around the pectoral girdle, thus quadrupedal. There is also a diagram showing a quadrupedal pose. |
| Tarjadia_ruthae | (Arcucci and Marsicano, 1998); (Sues, Desojo and Ezcurra, 2013) | OQ | Text - Sues et al (2013) suggests semi-aquatic lifestyle, hence OQ. |
| Tawa_hallae | (Sterling J Nesbitt *et al.*, 2009) | OB | Text – Theropod dinosaur, reasonably complete. Also has a diagram of a bipedal animal. |
| Teleocrater_combined | (Nesbitt *et al.*, 2017) | OQ | Diagram – based on a number of specimens, reconstructed in a quadrupedal pose. |
| Terrestrisuchus_gracilis | (Crush, 1984); (Irmis, Nesbitt and Sues, 2013) | OQ | Diagram – also discussed to be a cursorial terrestrial animal at length, but a mode is never explicitly stated in any literature |
| Ticinosuchus_ferox | (Krebs, 1965); (Lautenschlager and Desojo, 2011) | OQ | Text – “large, quadrupedal taxa (such as … *Ticinosuchus* …)”. |
| Trilophosaurus_buettneri | (Case, 1928); (Spielmann *et al.*, 2008) | OQ | Text – Arboreal, “used both its forelimbs and hindlimbs for propulsion”. Classifed as obligately quadrupedal. Also represented by a quadrupedal diagram. |
| Vancleavea_campi | (Long and Murry, 1995); (Nesbitt et al. 2009) | OQ | Text – aquatic, therefore OQ. |
| Velociraptor_mongoliensis | (Osborn, Kaisen and Olsen, 1924) | OB | Text – Theropod dinosaur, reconstructed as a biped multiple times. |
| Yarasuchus_deccanensis | (Sen, 2005) | FB | Text – “probably with a facultatively bipedal gait”. |
| Youngina_capensis | (Broom, 1914); (Gow, 1975) | OQ | Text – "This points towards a terrestrial quadrupedal existence”. |

Bibliography

Agassiz, L. (1844) *Monographie des poissons fossiles du vieux grés rouge : ou système Dévonien (Old red sandstone) des Iles Britanniques et de Russie / par L. Agassiz.* . Neuchâtel : A. Sonrel,.

Alcober, O. and Parrish, J. M. (1997) ‘A New Poposaurid from the Upper Triassic of Argentina’, *Journal of Vertebrate Paleontology*. Taylor & Francis, Ltd.The Society of Vertebrate Paleontology, 17, pp. 548–556. doi: 10.2307/4523835.

Allen, V. *et al.* (2010) ‘Functional specialization and ontogenetic scaling of limb anatomy in *Alligator mississippiensis*’, *Journal of Anatomy*. Blackwell Publishing Ltd, 216(4), pp. 423–445. doi: 10.1111/j.1469-7580.2009.01202.x.

Arcucci, A. and Marsicano, C. A. (1998) ‘A distinctive new archosaur from the middle triassic (los chañares formation) of argentina’, *Journal of Vertebrate Paleontology*. Taylor & Francis, Ltd.The Society of Vertebrate Paleontology, 18(1), pp. 228–232. doi: 10.1080/02724634.1998.10011046.

von Baczko, M. B. and Desojo, J. B. (2016) ‘Cranial anatomy and palaeoneurology of the archosaur *Riojasuchus tenuisceps* from the los colorados formation, La Rioja, Argentina’, *PLoS ONE*, 11(2), pp. 1–30. doi: 10.1371/journal.pone.0148575.

von Baczko, M. B. and Ezcurra, M. D. (2013) ‘Ornithosuchidae: a group of Triassic archosaurs with a unique ankle joint’, in S. J. Nesbitt, J. B. Desojo, R. B. I. (ed.) *Geological Society, London, Special Publications*. London: Geological Society, London, Special Publications, pp. 187–202. doi: 10.1144/SP379.4.

Barberena, M. C. (1982) ‘Uma nova espécie de Proterochampsa, *P. nodosa sp. nov.* do Triássico do Brasil’, *Anais da Academia Brasileira de Ciências*, 54, pp. 127–141.

Baron, M. G., Norman, D. B. and Barrett, P. M. (2016) ‘Postcranial anatomy of *Lesothosaurus diagnosticus* (Dinosauria: Ornithischia) from the Lower Jurassic of southern Africa: implications for basal ornithischian taxonomy and systematics’, *Zoological Journal of the Linnean Society*. Oxford University Press, 179(1), pp. 125–168. doi: 10.1111/zoj.12434.

Bassani, F. (1886) ‘Sui fossili e sull’eta degli schisti bituminosi Triasici di Besano in Lombardia’, *Atti della Società Italiana di Scienze Naturali*, 29, pp. 15–72.

Bates, K. *et al.* (2015) ‘Comments and corrections on 3D modeling studies of locomotor muscle moment arms in archosaurs’, *PeerJ*. PeerJ Inc., 3, p. e1272. doi: 10.7717/peerj.1272.

Bates, K. T. *et al.* (2012) ‘Computational modelling of locomotor muscle moment arms in the basal dinosaur *Lesothosaurus diagnosticus*: Assessing convergence between birds and basal ornithischians’, *Journal of Anatomy*, 220(3), pp. 212–232. doi: 10.1111/j.1469-7580.2011.01469.x.

Benton, M. J. (1990) ‘The Species of *Rhynchosaurus*, A Rhynchosaur (Reptilia, Diapsida) from the Middle Triassic of England’, *Philosophical Transactions of the Royal Society B: Biological Sciences*. The Royal Society, 328(1247), pp. 213–306. doi: 10.1098/rstb.1990.0114.

Bickelmann, C., Mü, J. and Reisz, R. R. (2009) ‘The enigmatic diapsid *Acerosodontosaurus piveteaui* (Reptilia: Neodiapsida) from the Upper Permian of Madagascar and the paraphyly of“ ‘younginiform’” reptiles’, *Canadian Journal of Earth Sciences*, 46, pp. 651–661. doi: 10.1139/E09-038.

Bonaparte, J. F. (1969) ‘Dos nuevas “faunas” de reptiles triasicos de Argentina’, *Gondwana Stratigraphy*, (2), pp. 282–306.

Breeden, B. T. (2016) *Observations on the osteology of* Scutellosaurus lawleri *Colbert, 1981 (Ornithischia: Thyreophora) on the basis on new specimens from the Lower Jurassic Kayenta Formation of Arizona*. University of Texas at Austin.

Bronzati, M. *et al.* (2017) ‘Endocast of the Late Triassic (Carnian) dinosaur *Saturnalia tupiniquim*: implications for the evolution of brain tissue in Sauropodomorpha’, *Scientific Reports*. Nature Publishing Group, 7(1), p. 11931. doi: 10.1038/s41598-017-11737-5.

Broom, R. (1903) ‘On a new reptile (*Proterosuchus fergusi*) from the Karroo Beds of Tarkastad, South Africa’, *Annals of the South African Museum*, 4, pp. 161–163.

Broom, R. (1905) ‘Notice of some new fosil reptiles from the Karroo Beds of South Africa’, *Records of the Albany Museum*, 1(5), p. 331:337.

Broom, R. (1913) ‘On the South-African Pseudosuchian *Euparkeria* and Allied Genera.’, *Proceedings of the Zoological Society of London*. Wiley/Blackwell (10.1111), 83(3), pp. 619–633. doi: 10.1111/j.1469-7998.1913.tb06148.x.

Broom, R. (1914) ‘A new thecodont reptile’, *Proceedings of the Zoological Society of London*, pp. 1072–1077.

Brown, B. (1933) ‘An ancestral crocodile’, *American Museum Novitates*. New York City : The American Museum of Natural History, 638, pp. 1–4.

Buckland, W. (1829) ‘On the Discovery of a New Species of Pterodactyle in the Lias at Lyme Regis.’, *Transactions of the Geological Society of London, series 2*. Geological Society of London, 3, pp. 217–222. doi: 10.1144/transgslb.3.1.217.

Camp, C. L. (1930) ‘A study of the phytosaurs with description of new matrerial from western North America’, *University of California Memoirs*, 10, pp. 1–174.

Casamiquela, R. M. (1960) ‘Notica preliminar sobre dos nuevos estagonolepoideos Argentinos.’, *Ameghiniana*, 2, pp. 3–9.

Case, E. C. (1928) ‘A cotylosaur from the Upper Triassic of western Texas’, *Jour Washington Acad Sci*. [Washington Academy of Sciences], 18, pp. 177–178.

Charig, A. J. and Reig, O. A. (1970) ‘The classification of the Proterosuchia’, *Biological Journal of the Linnean Society*. Wiley/Blackwell (10.1111), 2(2), pp. 125–171. doi: 10.1111/j.1095-8312.1970.tb01708.x.

Chatterjee, S. (1985) ‘*Postosuchus*, a New Thecodontian Reptile from the Triassic of Texas and the Origin of Tyrannosaurs’, *Philosophical Transactions of the Royal Society B: Biological Sciences*. The Royal Society, 309(1139), pp. 395–460. doi: 10.1098/rstb.1985.0092.

Chatterjee, S. (1993) ‘*Shuvosaurus*, a new theropod: an unusual theropod dinosaur from the Triassic of Texas’, *National Geographic Research and Exploration*, 9(3), pp. 274–285.

Chatterjee, S. (2001) ‘*Parasuchus Hislopi* Lydekker, 1885 (Reptilia, Archosauria): Proposed Replacement Of The Lectotype By A Neotype’, *The Bulletin of zoological nomenclature.* London,International Trust for Zoological Nomenclature., 58, pp. 34–36.

Clark, J. M. and Sues, H.-D. (2002) ‘Two new basal crocodylomorph archosaurs from the Lower Jurassic and the monophyly of the Sphenosuchia’, *Zoological Journal of the Linnean Society*. Blackwell Science Ltd, 136(1), pp. 77–95. doi: 10.1046/j.1096-3642.2002.00026.x.

Colbert, E. H. (1952) ‘A pseudosuchian reptile from Arizona’, *Bulletin of the American Museum of Natural History*. New York : [American Museum of Natural History], 99, pp. 565–592.

Colbert, E. H. (1970) ‘A saurischian dinosaur from the Triassic of Brazil’, *American Museum Novitates*. New York, N.Y. : American Museum of Natural History, 2045, pp. 1–39.

Colbert, E. H. (1981) *A primitive ornithischian dinosaur from the Kayenta Formation of Arizona*. Museum of North Arizona Press.

Colbert, E. H., Mook, C. C. and Brown, B. (1951) ‘The ancestral crocodilian *Protosuchus*’, *Bulletin of the AMNH*. New York : [American Museum of Natural History], 97, pp. 147–182.

Cope, E. D. (1887) ‘The dinosaurian genus *Coelurus*’, *American Naturalist*, 21, pp. 367–369.

Crompton, A. W. and Charig, A. J. (1962) ‘A new Ornithischian from the Upper Triassic of South Africa’, *Nature*. Nature Publishing Group, 196(4859), pp. 1074–1077. doi: 10.1038/1961074a0.

Crush, P. J. J. (1984) ‘A late upper Triassic sphenosuchid crocodilian from Wales’, *Palaeontology*, 27(1), pp. 131–157.

Currie, P. J. (1980) ‘A new younginid (Reptilia: Eosuchia) from the Upper Permian of Madagascar’, *Canadian Journal of Earth Sciences*. NRC Research Press Ottawa, Canada , 17(4), pp. 500–511. doi: 10.1139/e80-046.

Desojo, J. B. *et al.* (2013) ‘Aetosauria: a clade of armoured pseudosuchians from the Upper Triassic continental beds’, *Geological Society, London, Special Publications*. Geological Society of London, 379(1), pp. 203–239. doi: 10.1144/SP379.17.

Desojo, J. B., Ezcurra, M. D. and Schultz, C. L. (2011) ‘An unusual new archosauriform from the Middle-Late Triassic of southern Brazil and the monophyly of Doswelliidae’, *Zoological Journal of the Linnean Society*. Wiley/Blackwell (10.1111), 161(4), pp. 839–871. doi: 10.1111/j.1096-3642.2010.00655.x.

Dilkes, D. and Sues, H.-D. (2009) ‘Redescription and Phylogenetic Relationships of *Doswellia kaltenbachi* (Diapsida: Archosauriformes) from the Upper Triassic of Virginia’, *Journal of Vertebrate Paleontology*. Taylor & Francis, Ltd.The Society of Vertebrate Paleontology, 29, pp. 58–79. doi: 10.2307/20491069.

Dilkes, D. W. (1998) ‘The early Triassic rhynchosaur *Mesosuchus browni* and the interrelationships of basal archosauromorph reptiles’, *Philosophical Transactions of the Royal Society B: Biological Sciences*. The Royal Society, 353(1368), pp. 501–541. doi: 10.1098/rstb.1998.0225.

Dzik, J. (2003) ‘A Beaked Herbivorous Archosaur with Dinosaur Affinities from the Early Late Triassic of Poland’, *Journal of Vertebrate Paleontology*. Taylor & Francis, Ltd.The Society of Vertebrate Paleontology, 23(3), pp. 556–574. doi: 10.2307/4524350.

Evans, S. E. (1980) ‘The skull of a new eosuchian reptile from the Lower Jurassic of South Wales’, *Zoological Journal of the Linnean Society*. Oxford University Press, 70(3), pp. 203–264. doi: 10.1111/j.1096-3642.1980.tb00852.x.

Evans, S. E. (1981) ‘The postcranial skeleton of the Lower Jurassic eosuchian *Gephyrosaurus bridensis*’, *Zoological Journal of the Linnean Society*, 73(1), pp. 81–116. doi: 10.1111/j.1096-3642.1981.tb01580.x.

Evans, S. E. (1990) ‘The skull of *Cteniogenys*, a choristodere (Reptilia: Archosauromorpha) from the Middle Jurassic of Oxfordhsire’, *Zoological Journal of the Linnean Society*, 99, pp. 205–237.

Ewer, R. F. (1965) ‘The Anatomy of the Thecodont Reptile *Euparkeria capensis* Broom’, *Philosophical Transactions of the Royal Society B: Biological Sciences*. The Royal Society, 248(751), pp. 379–435. doi: 10.1098/rstb.1965.0003.

Ezcurra, M. D. (2016) ‘The phylogenetic relationships of basal archosauromorphs, with an emphasis on the systematics of proterosuchian archosauriforms’, *PeerJ*, 4, p. e1778. doi: 10.7717/peerj.1778.

Ezcurra, M. D. *et al.* (2018) ‘The osteology of the holotype of the early erythrosuchid *Garjainia prima* (Diapsida: Archosauromorpha) from the upper Lower Triassic of European Russia’, *Zoological Journal of the Linnean Society*, pp. 1–67. doi: 10.1093/zoolinnean/zly061.

Ezcurra, M. D. and Butler, R. J. (2015) ‘Taxonomy of the proterosuchid archosauriforms (Diapsida: Archosauromorpha) from the earliest Triassic of South Africa, and implications for the early archosauriform radiation’, *Palaeontology*. Edited by R. Benson. Wiley/Blackwell (10.1111), 58(1), pp. 141–170. doi: 10.1111/pala.12130.

Ezcurra, M. D., Butler, R. J. and Gower, D. J. (2013) ‘“Proterosuchia”: the origin and early history of Archosauriformes’, *Geological Society, London, Special Publications*. Geological Society of London, 379(1), pp. 9–33. doi: 10.1144/SP379.11.

Ezcurra, M. D., Montefeltro, F. and Butler, R. J. (2016) ‘The Early Evolution of Rhynchosaurs’, *Frontiers in Ecology and Evolution*. Frontiers, 3, p. 142. doi: 10.3389/fevo.2015.00142.

Ezcurra, M. D., Scheyer, T. M. and Butler, R. J. (2014) ‘The Origin and Early Evolution of Sauria: Reassessing the Permian Saurian Fossil Record and the Timing of the Crocodile-Lizard Divergence’, *PLoS ONE*. Edited by V. Ketmaier. Public Library of Science, 9(2), p. e89165. doi: 10.1371/journal.pone.0089165.

Farlow, J. O. *et al.* (2000) ‘Theropod Locomotion’, *American Zoologist*. Oxford University Press, 40(4), pp. 640–663. doi: 10.1093/icb/40.4.640.

Fechner, R. (2009) *Morphofunctional Evolution of the Pelvic Girdle and Hindlimb of Dinosauromorpha on the Lineage to Sauropoda*. Ludwigs Maximili-ans Universität.

Ferigolo, J. and Langer, M. C. (2007) ‘A Late Triassic dinosauriform from south Brazil and the origin of the ornithischian predentary bone’, *Historical Biology*, 19(1), pp. 23–33. doi: 10.1080/08912960600845767.

Flynn, J. *et al.* (2010) ‘A new species of *Azendohsaurus* (Diapsida: Archosauromorpha) from the Triassic Isalo Group of southwestern Madagascar: cranium and mandible’, *Palaeontology*. Wiley/Blackwell (10.1111), 53(3), pp. 669–688. doi: 10.1111/j.1475-4983.2010.00954.x.

Fraser, N. C. (1982) ‘A new rhynchocephalian from the British Upper Trias.’, *Palaeontology*, 25(4), pp. 709–725.

Fraser, N. C. and Rieppel, O. (2006) ‘A New Protorosaur (Diapsida) from the Upper Buntsandstein of the Black Forest, Germany’, *Journal of Vertebrate Paleontology*. Taylor & Francis, Ltd.The Society of Vertebrate Paleontology, 26(4), pp. 866–871. doi: 10.2307/4524638.

Fraser, N. C. and Walkden, G. M. (1984) ‘The postcranial skeleton of the Upper Triassic sphenodontid *Planocephalosaurus robinsonae*’, *Palaeontology*, 27(3), pp. 575–595.

Frigot, R. A. (2018) ‘ Pelvic musculature of Vectidraco daisymorrisae and consequences for pterosaur locomotion ’, *Geological Society, London, Special Publications*, 455(1), pp. 45–55. doi: 10.1144/sp455.7.

Galton, P. M. (1973) ‘On the anatomy and relationships of *Efraasia diagnostica* (Huene) n. gen., a prosauropod dinosaur (Reptilia: Saurischia) from the Upper Triassic of Germany’, *Paläontologische Zeitschrift*. Springer-Verlag, 47(3–4), pp. 229–255. doi: 10.1007/BF02985709.

Galton, P. M. (1978) ‘Fabrosauridae, the basal family of ornithischian dinosaurs (Reptilia: Ornithopoda)’, *Paläontologische Zeitschrift*. Springer-Verlag, 52(1–2), pp. 138–159. doi: 10.1007/BF03006735.

Gauthier, J. A. *et al.* (2011) ‘The bipedal stem crocodilian *Poposaurus gracilis*: Inferring function in fossils and innovation in archosaur locomotion’, *Bulletin of the Peabody Museum of Natural History*. Peabody Museum of Natural History, Yale University, 52(1), pp. 107–126. doi: 10.3374/014.052.0102.

Gervais, P. (1877) ‘Enumération de quelques ossements d’animaux vertébrés recueillis aux environs de Reims par M. Lemoine.’, *Journal de Zoologie*, 6, pp. 74–79.

Gilmore, C. W. (1928) ‘Fossil lizards of North America’, *Memoirs of the National Academy of Sciences*, 22(3), pp. 1–201.

Gottman-Quesada, A. and Sander, P. M. (2009) ‘A redescription of the early archosauromorph *Protosaurus speneri* Meter, and its phylogenetic relationships’, *Palaeontologica Abteilung A*, 287, pp. 123–220.

Gow, C. E. (1975) ‘The morphology and relationships of *Youngina capensis* Broom and *Prolacerta broomi* Parrington’, *Palaeontologica Africana*, 18, pp. 89–131.

Gow, C. E. and Kitching, J. W. (1988) ‘Early Jurassic crocodilomorphs from the Stormberg of South Africa’, *Neur Jahrbuch für Geologie und Paläontologie*, 9, pp. 517–536.

Gower, D. J. (1999) ‘The cranial and mandibular osteology of a new rauisuchian archosaur from the Middle Triassic of southern Germany’, *Stuttgarter Beiträge zur Naturkunde Serie B (Geologie und Paläontologie)* , 280, pp. 1–49.

Gower, D. J. *et al.* (2014) ‘A New Species of *Garjainia Ochev*, 1958 (Diapsida: Archosauriformes: Erythrosuchidae) from the Early Triassic of South Africa’, *PLoS ONE*. Edited by A. A. Farke. Public Library of Science, 9(11), p. e111154. doi: 10.1371/journal.pone.0111154.

Gower, D. J. and Schoch, R. R. (2009) ‘Postcranial anatomy of the rauisuchian archosaur *Batrachotomus kupferzellensis*’, *Journal of Vertebrate Paleontology*. Taylor & Francis Group, 29(1), pp. 103–122. doi: 10.1080/02724634.2009.10010365.

Hagen, C. J. *et al.* (2018) ‘Taphonomy, Geological Age, and Paleobiogeography of *Lotosaurus Adentus* (Archosauria: Poposauroidea) From the Middle-Upper Triassic Badong Formation, Hunan, China’, *Palaios*. Society for Sedimentary Geology, 33(3), pp. 106–124. doi: 10.2110/palo.2017.084.

Haughton, S. H. (1915) ‘A new thecodont from the Stormberg beds.’, *Annals of the South African Museum*, 12, pp. 98–105.

Hoffman, A. C. (1965) ‘On the discovery of a new thecodont from the Middle Beaufort Beds’, *Navorsinge van die Nasionale Museum Bloemfontein*, 2, pp. 33–40.

Huene, F. v. (1908) ‘Die Dinosaurier der europäischen Triasformation, mit Berücksichtigung der ausser-europäischen Vorkomnisse’, *Geologie und Paläontologie Abhandlungen*, 6, pp. 345–419.

Huene, F. v. (1942) ‘Lieferungen 3/4. Pseudosuchia, Saurischia, Rhynchosauridae und Schlussabschnitt’, in *Die Fossilen Reptilien des Südamerikanischen Gondwanalandes. Ergebnisse der Sauriergrabungen in Südbrasilien 1928/29*. München: C. H. Beck’sche Verlagsbuchhandlung, pp. 161–332.

Huxley, T. H. (1877) ‘The crocodilian remains found in the Elgin Sandstones, with remarks on the ichnites of Cummingstone’, *Memoirs of the Geological Survey of the United Kingdon, Monograph*, 3, pp. 1–52.

Irmis, R. B. *et al.* (2007) ‘A Late Triassic dinosauromorph assemblage from New Mexico and the rise of dinosaurs.’, *Science (New York, N.Y.)*. American Association for the Advancement of Science, 317(5836), pp. 358–61. doi: 10.1126/science.1143325.

Irmis, R. B., Nesbitt, S. J. and Sues, H.-D. (2013) ‘Early Crocodylomorpha’, *Geological Society, London, Special Publications*, 379, pp. 275–302.

Jalil, N.-E. (1997) ‘A new prolacertiform diapsid from the Triassic of North Africa and the interrelationships of the Prolacertiformes’, *Journal of Vertebrate Paleontology*. Taylor & Francis Group , 17(3), pp. 506–525. doi: 10.1080/02724634.1997.10010998.

Kimmig, J. (2013) ‘Possible secondarily terrestrial lifestyle in the European phytosaur *Nicrosaurus kapffi* (Late Triassic, Norian) -- A preliminary study’, *Bulletin of the New Mexico Museum of Natural History and Science*, 61, pp. 306–312.

Krebs, B. (1965) ‘*Ticinosuchus ferox n. g. n. sp.* Ein neuer Pseudosuchier aus der Trias des Monte San Giorgio’, *Schweizerische Paläontologische Abhandlungen*, 81, pp. 1–140.

Kubo, T. and Kubo, M. O. (2012) ‘Associated evolution of bipedality and cursoriality among Triassic archosaurs: a phylogenetically controlled evaluation’, *Paleobiology*. The Paleontological Society , 38(03), pp. 474–485. doi: 10.1666/11015.1.

Lane, H. H. (1945) ‘New Mid-Pennsylvanian Reptiles from Kansas’, *Transactions of the Kansas Academy of Science (1903-)*. Kansas Academy of Science, 47(3), p. 381. doi: 10.2307/3625454.

Langer, M. C. *et al.* (1999) ‘A sauropodomorph dinosaur from the Upper Triassic (Carman) of southern Brazil’, *Comptes Rendus de l’Académie des Sciences - Series IIA - Earth and Planetary Science*. Elsevier Masson, 329(7), pp. 511–517. doi: 10.1016/S1251-8050(00)80025-7.

Langer, M. C. *et al.* (2010) ‘On *Fodonyx spenceri* and a new rhynchosaur from the Middle Triassic of Devon’, *Journal of Vertebrate Paleontology*. Taylor & Francis Group, 30(6), pp. 1884–1888. doi: 10.1080/02724634.2010.521901.

Langer, M. C. and Ferigolo, J. (2013) ‘The Late Triassic dinosauromorph *Sacisaurus agudoensis* (Caturrita Formation; Rio Grande do Sul, Brazil): anatomy and affinities’, *Geological Society, London, Special Publications*, 379, pp. 353–392.

Lautenschlager, S. and Desojo, J. B. (2011) ‘Reassessment of the Middle Triassic rauisuchian archosaurs *Ticinosuchus ferox* and *Stagonosuchus nyassicus*’, *Palaontologische Zeitschrift*. Springer-Verlag, 85(4), pp. 357–381. doi: 10.1007/s12542-011-0105-1.

Lautenschlager, S. and Rauhut, O. W. M. M. (2015) ‘Osteology of *Rauisuchus tiradentes* from the Late Triassic (Carnian) Santa Maria Formation of Brazil, and its implications for rauisuchid anatomy and phylogeny’, *Zoological Journal of the Linnean Society*. Oxford University Press, 173(1), pp. 55–91. doi: 10.1111/zoj.12196.

Li, C. *et al.* (2006) ‘An unusual archosaurian from the marine Triassic of China’, *Naturwissenschaften*. Springer-Verlag, 93(4), pp. 200–206. doi: 10.1007/s00114-006-0097-y.

Liparini, A. and Schultz, C. L. (2013) ‘A reconstruction of the thigh musculature of the extinct pseudosuchian *Prestosuchus chiniquensis* from the *Dinodontosaurus* Assemblage Zone (Middle Triassic Epoch), Santa Maria 1 Sequence, southern Brazil’, *Geological Society, London, Special Publications*. Geological Society of London, 379(1), pp. 441–468. doi: 10.1144/SP379.20.

Long, R. A. and Murry, P. A. (1995) *Late Triassic (Carnian and Norian) tetrapods from the southwestern United States*, *New Mexico Museum of Natural History and Science Bulletin*.

Lydekker, R. (1885) ‘The Reptilia & Amphibia of the Maleria & Denwa Groups’, *Memoirs of the Geological Survey of India. Palaeontologica Indica, Servies IV. Indian Pretertiary Vertebrata*, 1(5), pp. 1–38.

Mallison, H. (2010) ‘The Digital *Plateosaurus* I : Body Mass , Mass Distribution and Posture Assessed Using CAD and CAE on a Digitally Mounted Complete Skeleton’, *Palaeontologia Electronica*, 2(2), pp. 1–26.

Marsh, O. C. (1877) ‘Notice of new dinosaurian reptiles from the Jurassic formation’, *American Journal of Science*. American Journal of Science, s3-14(84), pp. 514–516. doi: 10.2475/ajs.s3-14.84.514.

Matsumoto, R. and Evans, S. E. (2010) ‘Choristoderes and the freshwater assemblages of Laurasia’, *Journal of Iberian Geology*, 36(2), pp. 253–274.

Mehl, M. G. (1915) ‘*Poposaurus gracilis*, a New Reptile from the Triassic of Wyoming’, *The Journal of Geology*. The University of Chicago Press, 23(6), pp. 516–522. doi: 10.2307/30067173.

Mehl, M. G. (1928) ‘*Pseudopalatus pristinus*, a new genus and species of phytosaurs from Arizona.’, *The University of Missouri Studies*, 3, pp. 7–22.

Meyer, H. v. (1832) *Palaeologica zur Geschichte der Erde und ihrer Geschöpfe*. Frankfurt am Main: Verlag von Siegmund Schmerber.

Meyer, H. v. (1837) ‘Mittheilungen, an Professor Bronn gerichtet.’, *Neues Jahrbuch für Mineralogie, Geognosie, Geologie und Petrefaktenkunde*, 1837, pp. 314–317.

Meyer, H. v. (1860) ‘Briefliche Mittheilug an Prof. Bronn.’, *Neues Jahrbuch für Mineralogie, Geognosie, Geologie, und Petrefakten-Kunde*, pp. 556–560.

Nesbitt, S. J. (2005) ‘Osteology of the Middle Triassic pseudosuchian archosaur *Arizonasaurus babbitti*’, *Historical Biology*. Taylor & Francis Group, 17(1–4), pp. 19–47. doi: 10.1080/08912960500476499.

Nesbitt, S. J. (2007) ‘The anatomy of *Effigia okeeffeae* (Archosauria, Suchia), theropod-like convergence, and the distribution of related taxa’, *Bulletin of the American Museum of Natural History*. New York, NY : American Museum of Natural History, 302.

Nesbitt, Sterling J *et al.* (2009) ‘A complete skeleton of a Late Triassic saurischian and the early evolution of dinosaurs.’, *Science*. American Association for the Advancement of Science, 326(5959), pp. 1530–3. doi: 10.1126/science.1180350.

Nesbitt, Sterling J., Irmis, R. B., *et al.* (2009) ‘Hindlimb osteology and distribution of basal dinosauromorphs from the Late Triassic of North America’, *Journal of Vertebrate Paleontology*. Taylor & Francis Group , 29(2), pp. 498–516. doi: 10.1671/039.029.0218.

Nesbitt, Sterling J., Stocker, M. R., *et al.* (2009) ‘The osteology and relationships of *Vancleavea campi* (Reptilia: Archosauriformes)’, *Zoological Journal of the Linnean Society*. Blackwell Publishing Ltd, 157(4), pp. 814–864. doi: 10.1111/j.1096-3642.2009.00530.x.

Nesbitt, S. J. *et al.* (2010) ‘Ecologically distinct dinosaurian sister group shows early diversification of Ornithodira’, *Nature*. Nature Publishing Group, 464(7285), pp. 95–98. doi: 10.1038/nature08718.

Nesbitt, S. J. *et al.* (2013) ‘Rauisuchia’, *Geological Society, London, Special Publications*. Geological Society of London, 379(1), pp. 241–274. doi: 10.1144/SP379.1.

Nesbitt, S. J. *et al.* (2014) ‘A new archosaur from the Manda beds (Anisian, Middle Triassic) of southern Tanzania and its implications for character state optimizations at Archosauria and Pseudosuchia’, *Journal of Vertebrate Paleontology*. Taylor & Francis, 34(6), pp. 1357–1382. doi: 10.1080/02724634.2014.859622.

Nesbitt, S. J. *et al.* (2015) ‘Postcranial Osteology of *Azendohsaurus madagaskarensis* (?Middle to Upper Triassic, Isalo Group, Madagascar) and its Systematic Position Among Stem Archosaur Reptiles’, *Bulletin of the American Museum of Natural History*. American Museum of Natural History, Library-Scientific Publications Central Park West at 79th St., New York, NY 10024, 398, pp. 1–126. doi: 10.1206/amnb-899-00-1-126.1.

Nesbitt, S. J. *et al.* (2017) ‘The earliest bird-line archosaurs and the assembly of the dinosaur body plan’, *Nature*. Nature Research, 544(7651), pp. 484–487. doi: 10.1038/nature22037.

Nesbitt, S. J., Irmis, R. B. and Parker, W. G. (2007) ‘A critical re‐evaluation of the Late Triassic dinosaur taxa of North America’, *Journal of Systematic Palaeontology*. Taylor & Francis Group, 5(2), pp. 209–243. doi: 10.1017/S1477201907002040.

Nesbitt, S. J. and Norell, M. A. (2006) ‘Extreme convergence in the body plans of an early suchian (Archosauria) and ornithomimid dinosaurs (Theropoda).’, *Proceedings. Biological sciences*. The Royal Society, 273(1590), pp. 1045–8. doi: 10.1098/rspb.2005.3426.

Nopcsa, F. (1930) ‘Notizen über *Macrochemus bassanii nov. gen. et spec.*’, *Centralblatt für Mineralogie, Geologie und Paläontologie, Abteilung B: Geologie und Paläontologie*, 1930, pp. 252–255.

Ochev, V. G. (1958) ‘[New data concerning the pseudosuchians of the USSR]’, *Doklady AN SSSR*, 123, pp. 331–337.

Osborn, H. F., Kaisen, P. C. and Olsen, G. (1924) *Three new Theropoda, Protoceratops zone, central Mongolia*. New York City: The American Museum of Natural History.

Owen, R. (1842) ‘Description of an Extinct Lacertilian Reptile, *Rhynchosaurus articeps*, Owen, of which the Bones and Foot-prints characterize the Upper New Red Sandstone at Grinshill, near Shrewsbury’, *Transactions of the Cambridge Philosophical Society*, 7(3), pp. 355–369.

Padian, K. (2008) ‘Were pterosaur ancestors bipedal or quadrupedal?: Morphometric, functional, and phylogenetic considerations’, *Zitteliana Reihe B: Abhandlungen der Bayerischen Staatssammlung fur Palaontologie und Geologie*, 28B, pp. 21–28.

Parker, W. G. and Nesbitt, S. J. (2013) ‘Cranial remains of *Poposaurus gracilis* (Pseudosuchia: Poposauroidea) from the Upper Triassic, the distribution of the taxon, and its implications for poposauroid evolution’, *Geological Society, London, Special Publications*. Geological Society of London, 379(1), pp. 503–523. doi: 10.1144/SP379.3.

Parrington, F. R. (1935) ‘On *Prolacerta broomi, gen. et sp. n.* and the origin of lizards’, *Annals and Magazine of Natural History*, 16, pp. 197–205.

Peabody, F. E. (1952) ‘*Petrolacosaurus kansensis* Lane, a Pennsylvanian reptile from Kansas’, *The University of Kansas Paleontological Contributions, Series Vertebrata*, 1, pp. 1–41.

Peyer, K. *et al.* (2008) ‘A new suchian archosaur from the Upper Triassic of North Carolina’, *Journal of Vertebrate Paleontology*, 28(2), pp. 363–381. doi: 10.1671/0272-4634(2003)023[0329:ANCAFT]2.0.CO;2.

Piechowski, R. and Dzik, J. (2010) ‘The axial skeleton of *Silesaurus opolensis*’, *Journal of Vertebrate Paleontology*. Taylor & Francis Group, 30(4), pp. 1127–1141. doi: 10.1080/02724634.2010.483547.

Pontzer, H., Allen, V. and Hutchinson, J. R. (2009) ‘Biomechanics of running indicates endothermy in bipedal dinosaurs’, *PLoS ONE*. Public Library of Science, 4(11), p. e7783. doi: 10.1371/journal.pone.0007783.

Reig, O. A. (1959) ‘Primeros datos descriptivos sobre nuevos arcosaurios del Triásico de Ischigualasto (San Juan, Argentina)’, *Rev. Asoc. Geol. Argentina*, 13(4), pp. 257–270.

Reig, O. A. (1963) ‘La presencia de dinosaurios saurisquios en los “Estratos de Ischigualasto” (Mesotriasico Superior) de las provincias de San Juan y La Rioja (República Argentina)’, *Ameghiniana*, 3(1), pp. 3–20.

Renesto, S. (2005) ‘A new specimen of *Tanystropheus* (Reptilia Protorosauria) from the Middle Triassic of Switzerland and the ecology of the genus’, *Rivista Italiana di Paleontologia e Stratigrafia*, 111(3), pp. 377–394. doi: 10.13130/2039-4942/6327.

Rieppel, O. (1989) ‘The Hind Limd of *Macrocnemus bassanii* (Nopcsa) (Reptilia, Diapsida): Development and Functional Anatomy’, *Journal of Vertebrate Paleontology*. Taylor & Francis, Ltd.The Society of Vertebrate Paleontology, pp. 373–387. doi: 10.2307/4523279.

Romer, A. S. (1971a) ‘The Chanares (Argentina) Triassic reptile fauna. X. Two new but incompletely known long-limbed pseudosuchians’, *Breviora*, 378, pp. 1–10. doi: 10.5962/bhl.part.6285.

Romer, A. S. (1971b) ‘The Chanares (Argentina) Triassic reptile fauna. XI. Two new long-snouted thecodonts, *Chanaresuchus* and *Gualosuchus*’, *Breviora.* Museum of Comparative Zoology, Harvard University, 379, pp. 1–22.

Romer, A. S. (1972a) ‘The Chañares (Argentina) Triassic Reptile Fauna. XII. The Postcranial Skeleton Of The Thecodont *Chanaresuchus*’, *Breviora*, 385, pp. 1–21.

Romer, A. S. (1972b) ‘The Chanares (Argentina) Triassic reptile fauna. XIII. An early ornithosuchid pseudosuchian, *Gracilisuchus stipanicicorum, gen. et sp. nov*’, *Breviora*, 389, pp. 1–24. doi: 10.5962/bhl.part.8674.

Romer, A. S. (1972c) ‘The Chanares (Argentina) Triassic reptile fauna. XV. Further remains of the thecodonts *Lagerpeton* and *Lagosuchus*’, *Breviora*, 394, pp. 1–7.

Sawin, H. J. (1947) ‘The Pseudosuchian Reptile *Typothorax meadei*’, *Journal of Paleontology*. SEPM Society for Sedimentary Geology, 21(3), pp. 201–238. doi: 10.2307/1299332.

Schachner, E. R., Manning, P. L. and Dodson, P. (2011) ‘Pelvic and hindlimb myology of the basal archosaur *Poposaurus gracilis* (archosauria: Poposauroidea)’, *Journal of Morphology*. Wiley Subscription Services, Inc., A Wiley Company, 272(12), pp. 1464–1491. doi: 10.1002/jmor.10997.

Schoch, R. R. (2007) ‘Osteology of the small archosaur *Aetosaurus*  from the Upper Triassic of Germany’, *Neues Jahrbuch für Geologie und Paläontologie - Abhandlungen*, 246(1), pp. 1–35. doi: 10.1127/0077-7749/2007/0246-0001.

Schoch, R. R. and Sues, H.-D. (2014) ‘A new archosauriform reptile from the Middle Triassic (Ladinian) of Germany’, *Journal of Systematic Palaeontology*. Taylor & Francis, 12(1), pp. 113–131. doi: 10.1080/14772019.2013.781066.

Sen, K. (2003) ‘*Pamelaria dolichotrachela*, a new prolacertid reptile from the Middle Triassic of India’, *Journal of Asian Earth Sciences*. Pergamon, 21(6), pp. 663–681. doi: 10.1016/S1367-9120(02)00110-4.

Sen, K. (2005) ‘A new rauisuchian archosaur from the Middle Triassic of India’, *Palaeontology*. Wiley/Blackwell (10.1111), 48(1), pp. 185–196. doi: 10.1111/j.1475-4983.2004.00438.x.

Sennikov, A. G. (1988) ‘Novyye rauizukhidy iz triasa yevropeyskoy chasti SSSR’, *Paleontol. Zhurn*, 1990(2), pp. 124–128.

Sereno, P. C. *et al.* (1993) ‘Primitive dinosaur skeleton from Argentina and the early evolution of Dinosauria’, *Nature*. Nature Publishing Group, 361(6407), pp. 64–66. doi: 10.1038/361064a0.

Sereno, P. C. and Arcucci, A. B. (1994) ‘Dinosaurian precursors from the Middle Triassic of Argentina: *Marasuchus lilloensis* , gen. nov.’, *Journal of Vertebrate Paleontology*. Taylor & Francis, Ltd.The Society of Vertebrate Paleontology, pp. 53–73. doi: 10.1080/02724634.1994.10011538.

Sereno, P. C., Mar Inez, R. N. and Alcober, O. A. (2013) ‘Osteology Of *Eoraptor Lunensis* (Dinosauria, Sauropodomorpha)’, *Society of Vertebrate Paleontology Memoir 12 Journal of Vertebrate Paleontology*, 32(6), pp. 83–179.

Sereno, P. C. and Novas, F. E. (1992) ‘The complete skull and skeleton of an early dinosaur.’, *Science (New York, N.Y.)*. American Association for the Advancement of Science, 258(5085), pp. 1137–40. doi: 10.1126/science.258.5085.1137.

Simmons, D. J. (1965) ‘The non-therapsid reptiles of the Lufeng Basin, Yunnan, China’, *Fieldiana: Geology*. Chicago : Chicago Natural History Museum Press, 15(1), pp. 1–116. doi: 10.5962/bhl.title.5426.

Spielmann, J. A. *et al.* (2008) ‘The Late Triassic Archosauromorph *Trilophosaurus*’, *New Mexico Museum of Natural History and Science Bulletin*, 43, pp. 1–177.

Stocker, M. R. and Butler, R. J. (2013) ‘Phytosauria’, *Geological Society, London, Special Publications*. Geological Society of London, 379(1), pp. 91–117. doi: 10.1144/SP379.5.

Sues, H.-D., Desojo, J. B. and Ezcurra, M. D. (2013) ‘Doswelliidae: a clade of unusual armoured archosauriforms from the Middle and Late Triassic’, *Geological Society, London, Special Publications*. Geological Society of London, 379(1), pp. 49–58. doi: 10.1144/SP379.13.

Sulej, T. (2005) ‘A new rauisuchian reptile (Diapsida: Archosauria) from the Late Triassic of Poland’, *Journal of Vertebrate Paleontology*. Taylor & Francis Group, 25(1), pp. 78–86. doi: 10.1671/0272-4634(2005)025[0078:ANRRDA]2.0.CO;2.

Trotteyn, M. J., Arcucci, A. B. and Raugust, T. (2013) ‘Proterochampsia: an endemic archosauriform clade from South America’, *Geological Society, London, Special Publications*. Geological Society of London, 379(1), pp. 59–90. doi: 10.1144/SP379.23.

Trotteyn, M. J. and Ezcurra, M. D. (2014) ‘Osteology of *Pseudochampsa ischigualastensis gen. et comb. nov.* (Archosauriformes: Proterochampsidae) from the Early Late Triassic Ischigualasto Formation of Northwestern Argentina’, *PLoS ONE*. Edited by P. Dodson. Public Library of Science, 9(11), p. e111388. doi: 10.1371/journal.pone.0111388.

Walker, A. D. (1990) ‘A Revision of *Sphenosuchus acutus* Haughton, a Crocodylomorph Reptile from the Elliot Formation (Late Triassic or Early Jurassic) of South Africa’, *Philosophical Transactions of the Royal Society B: Biological Sciences*. The Royal Society, 330(1256), pp. 1–120. doi: 10.1098/rstb.1990.0185.

Watson, D. M. S. (1912a) ‘*Eosuchus colletti, gen. et spec. nov*’, *Records of the Albany Museum*, 2, pp. 298–299.

Watson, D. M. S. (1912b) ‘*Mesosuchus Brownii*, Gen. Et Spec. Nov’, *Records of the Albany Museum*, 2, pp. 296–297.

Weems, R. E. (1980) ‘An Unusual Newly Discovered Archosaur from the Upper Triassic of Virginia, U. S. A.’, *Transactions of the American Philosophical Society*. American Philosophical Society, 70(7), p. 1. doi: 10.2307/1006472.

Weinbaum, J. C. (2013) ‘Postcranial skeleton of *Postosuchus kirkpatricki* (Archosauria: Paracrocodylomorpha), from the Upper Triassic of the United States’, *Geological Society, London, Special Publications*, 379(1), pp. 525–553. doi: 10.1144/SP379.7.

Welles, S. P. (1947) ‘Vertebrates from the Upper Moenkopi Formation of northern Arizona’, *University of California Publications in Geological Sciences*, 27(7), pp. 241–294.

Welles, S. P. (1954) ‘New Jurassic dinosaur from the Kayenta formation of Arizona’, *GSA Bulletin*. GeoScienceWorld, 65(6), pp. 591–598. doi: 10.1130/0016-7606(1954)65[591:njdftk]2.0.co;2.

Witton, M. P. (2015) ‘Were early pterosaurs inept terrestrial locomotors?’, *PeerJ*. PeerJ Inc., 3, p. e1018. doi: 10.7717/peerj.1018.

Wu, X.-C. and Chatterjee, S. (1993) ‘*Dibothrosuchus elaphros*, a Crocodylomorph from the Lower Jurassic of China and the Phylogeny of the Sphenosuchina’, *Journal of Vertebrate Paleontology*. Taylor & Francis, Ltd.The Society of Vertebrate Paleontology, 13(1), pp. 58–89. doi: 10.2307/4523486.

Wu, X. (1986) ‘A new species of *Dibothrosuchus* from Lufeng Basin’, *Vertebrata PalAsiatica*, 24, pp. 43–62.

Young, C. C. (1936) ‘On a new *Chasmatosaurus* from Sinkiang’, *Bulletin of the Geological Society of China*. Wiley/Blackwell (10.1111), 15(3), pp. 291–311. doi: 10.1111/j.1755-6724.1936.mp15003003.x.

Young, C. C. (1964) ‘The pseudosuchians in China’, *Palaeontologia Sinica*, 19, pp. 1–205.

Zambelli, R. (1973) ‘*Eudimorphodon ranzii gen. nov., sp. nov.*, uno pterosauro triassico’, *Rendiconti Scienze di Instituto Lombardo, B*, 107, p. 27.

Zhang, F.-K. (1975) ‘A new thecodont *Lotosaurus*, from Middle Triassic of Hunan’, *Vertebrata PalAsiatica*, 13, pp. 144–147.
